# Evaluation of the quality of meat and carcasses from sheep fed diets containing three types of oils

**DOI:** 10.3389/fvets.2023.1103516

**Published:** 2023-07-06

**Authors:** Thays Syntya Antunes da Costa, Jamile Andréa Rodrigues da Silva, Cristian Faturi, André Guimarães Maciel e Silva, Aníbal Coutinho do Rêgo, Edwana Mara M. Monteiro, Juliana Cristina de Castro Budel, Vinícius Costa Gomes de Castro, Antônio Vinicius Corrêa Barbosa, Welligton Conceição da Silva, José de Brito Lourenço-Junior

**Affiliations:** ^1^Postgraduate Program in Animal Science (PPGCAN), Institute of Veterinary Medicine, Brazilian Agricultural Research Corporation (EMBRAPA), Federal University of Para (UFPA), Federal Rural University of the Amazon (UFRA), Castanhal, Brazil; ^2^Institute of Animal Health and Production, Federal Rural University of the Amazônia (UFRA), Belém, Brazil; ^3^Institute of Medicine Veterinary, Universidade da Amazônia (UNAMA), Belém, Brazil; ^4^Faculty of Formation and Field Development, Federal University of Para (UFPA), Abaetetuba, Brazil; ^5^Postgraduate Program in Animal Health and Production in the Amazon (PPGSPAA), Federal Rural University of the Amazon (UFRA), Belém, Brazil; ^6^Cyberspace Institute, Federal Rural University of the Amazon (UFRA), Belém, Brazil

**Keywords:** lipid diets, residual oil, sheep, palm oil, characteristics of carcasses

## Abstract

The objective of this research was to evaluate the quantitative and qualitative characteristics of the carcass and meat of lambs fed with different types of oil. Thirty male, uncastrated, mixed breed (Santa Inês × Dorper) sheep were used in this experiment and were distributed in random blocks with three treatments and 10 replicates per treatment, where each animal represents a replicated experimental unit. The three treatments were the following experimental diets: soybean oil in natura; soybean oil after use in frying, and palm oil (*Elaeis guineensis*). The oils were incorporated into the sheep diets at a level of 4%. Carcasses were evaluated for hot and cold yield, loss due to cooling, finish, conformation, internal fat concentration, morphometric measurements, tissue composition, and commercial cuts. The addition of soybean oil in natura, residual soybean oil from frying, and palm oil to the sheep diets did not alter any of the evaluated characteristics, which therefore can be interpreted as favoring the use of any of these three oils as a supplement to their diet, and the three oils imparted good characteristics to the carcasses and the meat.

## 1. Introduction

The growing interest in intensifying the finishing process of lambs in confinement is aimed at accelerating the commercialization and production processes of carcasses and meat with high yield. However, this type of management requires greater investment in infrastructure, equipment, feed, and labor. Concerns about increasing production costs require research into alternative feed sources that have good nutritional value at a low cost (1).

The use of vegetable oils in ruminant feed can have positive effects such as a reduction in the production of methane and the concentration of N-NH3 in the rumen, an increase in the efficiency of microbial synthesis, an influence on physiological processes, alterations in the profile of fatty acids and their derivatives, and an increase in the overall energy concentration of the diet (1). However, vegetable oils should be used with caution because high levels of foods rich in unsaturated fatty acids (above 4% dietary DM) are toxic to cellulolytic microorganisms in the rumen (2); by impairing the degradation of fiber in the rumen, which lowers DM intake and performance. In addition, different studies have sought to evaluate the effects of oils on sheep (3–6).

The high cost of vegetable oils restricts their use in sheep diets. However, an alternative could be the use of residual cooking oil from frying. Another alternative for inclusion in ruminant diets is palm oil (*Elaeis guineenses*), especially since the state of Pará is the largest producer in Brazil (7). In the meat production system, the quantitative and qualitative characteristics of the carcass are of fundamental importance since these are directly related to the final product (8).

In this context, there exists the possibility of substituting conventional feed sources for residual cooking oil from frying and palm oil, which represents an alternative to minimize environmental impact in addition to increasing the overall energy concentration of the sheep’s diet, influencing the variables of interest of the carcass and meat, and reducing production costs. Cooking oil after its use is usually discarded, but in this research, we sought to use it as a source of food for animals. Given this background, this work aimed to evaluate the influence of the diet of lambs fed with different types of oil on the quantitative and qualitative characteristics of the carcass and meat.

## 2. Materials and methods

### 2.1. Ethics committee

The experiment was conducted in accordance with the ethical standards established by the Committee for the Ethical Treatment of Animals in Experimentation of the Federal Rural University of the Amazon (UFRA) and was approved by the same committee under protocol number 005/2013.

### 2.2. Experimental area

This experiment was conducted in Castanhal, Pará, Brazil (1°17′S and 47°55′W), in a hot and humid climate with climate type Am according to the Köppen classification, with an average annual rainfall of 2.770 mm/year, an average annual temperature of 26.8°C, and an average annual relative humidity of 85%. Laboratory analyses were carried out at the Animal Nutrition Laboratory of the Universidade Federal Rural da Amazônia (UFRA), at the Animal Nutrition Laboratory of the Universidade Federal do Pará (UFPA), *Campus,* Castanhal, Pará and at the Physical–Chemical Meat Analysis Laboratory of the Instituto Federal do Pará (IFPA/Campus Castanhal).

### 2.3. Animals and experimental treatment

A total of 30 male, uncastrated, mixed-breed (Santa Inês × Dorper) lambs with an initial average body mass of 21 ± 3 kg were used in this experiment. The animals were identified with ear tags and distributed in random blocks of three treatments with ten replicates per treatment, with each animal representing a replicated experimental unit. The animals were randomly assigned to individual wooden stalls with a cement floor and a total area of 1.48 m^2^ (1.52 m × 0.97 m), with access to individual feeders and water sources, and the feed was weighed in order to track consumption and the amount left over after feeding.

The stalls were in a covered hangar that provided protection from rain and direct sunlight, with lateral openings for air circulation, thus maintaining a well-ventilated internal environment and promoting animal welfare. The experimental period lasted 80 days, 10 of which were used for animal adaptation to the diets and facilities. Before the experimental period, the area of the stalls was checked for zootechnical and sanitary abnormalities in order to control endo-and ectoparasites. As a prophylactic measure for anthelmintic control, ivermectin was administered subcutaneously at a dose of 0.5 mL/25 kg of live mass, and the animals received vitamins A, D, E, and B12.

The three experimental diets used were: 1. control, *soybean oil in natura*; 2. soybean oil after frying; and 3. palm oil (*Elaeis guineensis*). The oils were added to the sheep diets at a level of 4% of the dry weight of the feed diet (Table 1). The chemical composition of the feed is presented in Table 2. The residual cooking oil was collected after being used for frying for approximately 6 h per day for 3 days, which corresponds to a total of 18 h of exposure to the gas fire, and was obtained from a local small business that exclusively sells freshly fried French fries and whose operations were certified as complying with Standard 8/2004 of the Ministry of Agriculture, Livestock, and Food Supply, which prohibits the contamination of products of non-animal origin with products of animal origin.

**Table 1:** Chemical and centesimal compositions of the experimental diets.

Consumables	Centesimal composition (%)
Silage	40.00
Corn	23.00
Soy bran	16.30
Wheat bran	15.00
Oil	4.00
Urea	1.10
Calcitic limestone	0.60
Nutrients	Chemical composition
DM^1^	70.45
OM^2^	94.77
MM^2^	5.23
CP^2^	17.98
NDIN^3^	21.06
ADIN^3^	5.67
EE^2^	6.66
TC^2^	71.52
NDF_AP_^2^	37.77
NFC^2^	29.29
ADF^2^	19.66
LIG^2^	3.85

^1^Percentage of natural matter, ^2^percentage of dry matter, and ^3^percentage of total nitrogen.

**Table 2:** Chemical composition of feed.

Composition	Ingredient			
	Silage	Ground corn	Soy bran	Wheat bran
DM^1^	19.44	89.93	89.15	90.18
OM^2^	93.41	98.38	92.50	93.34
MM^2^	6.59	1.62	7.50	6.66
CP^2^	10.30	10.26	40.31	12.22
NDIN^3^	25.20	12.38	13.70	39.30
ADIN^3^	10.32	1.07	2.56	5.89
EE^2^	3.06	2.90	2.46	2.45
TC^2^	80.05	85.22	49.74	78.66
NDF^2^	67.42	20.44	16.16	52.91
NDF_CP_^2^	61.94	19.04	10.32	46.26
NFC^2^	12.63	64.78	33.58	25.75
NFC_CP_^2^	18.11	66.17	39.42	32.40
ADF^2^	40.30	0.96	7.31	14.15
ADF_CP_^2^	39.31	0.85	6.13	13.07
LIG^2^	5.77	2.29	2.94	3.58

^1^%natural material, ^2^values in % of dry material, ^3^values in % of total nitrogen.Estimated using the NRC equation (9).

DM, Dry material; OM, Organic matter; MM, Mineral matter; CP, Crude protein; NDIN, Neutral detergent insoluble nitrogen; ADIN, Acid detergent insoluble nitrogen; EE, Ether extract; TC, Total carbohydrates; NDF, Neutral detergent fiber; NDF_CP_, corrected for ash and protein; NFC, Non-fibrous carbohydrate; NFC_CP_, NFC corrected for ash and protein; ADF, Acid detergent fiber; LIG, Lignin.

Fatty acid composition (Table 3) was determined by methyl ester conversion according to AOCS method Ce 2–66 and analysis with a CP3380 gas chromatograph (Varian Inc., United States) connected to a Varian CP-Sil 88 capillary column (60 m × 0.25 mm) and coupled to a flame ionization detector. Injections were performed in duplicate. Peaks were identified by comparing retention times. The calibration curve was determined using a mixture of standard fatty acids (Nucheck 74X). The area of each peak and retention times were calculated using Varian Star^©^ 3.4.1 software, and the results were expressed as percentages (10).

**Table 3:** Fatty acid composition of the oils in the experimental diets.

Fatty acids	Percentage (g kg^−1^)		
	Soybean oil	Yellow grease	Palm oil
Lauric acid (C12:0)	–	–	2.6
Myristic acid (C14:0)	–	–	5.3
Palmitic acid (C16:0)	11.78	15.35	35.69
Palmitoleic acid (C16:1)	–	–	1.4
Stearic acid (C18:0)	3.79	5.03	5.22
Oleic acid (C18:1)	30.68	30.44	47.38
Linoleic acid (C18:2)	46.64	43.03	9.89
Linolenic acid (C18:3)	4.22	3.80	2.4
Arachidic acid (C20:0)	8.8	5.2	6.4
Behenic acid (C22:0)	1.13	1.13	–
Erucic acid (C22:1)	8.8	–	–
Lignoceric acid (C24:0)	–	6.9	–
Saturated	17.58	22.03	42.34
Unsaturated	82.42	77.96	57.65

The diets were formulated to promote an average daily weight gain of 200 g/day, according to the NRC method (11), and to be isoproteic with a volume:concentration ratio of 40:60. They consisted of silage made from elephant grass (*Pennisetum purpureum*, Schum.) and ground corn, soybean, and wheat bran, the oils from the three treatments, calcitic limestone, and urea. The animals were fed twice a day, at 08:00 and at 16:00, with enough feed to ensure that 10% was left over after each meal, and they had unlimited access to water and salt. After this period, the animals were slaughtered following a 12-h fast.

The elephant grass was harvested at approximately 70 days of age and processed using a vegetation mulcher that generated a 10 mm mulch, and the mulched grass was stored in silo bags. Wheat bran was mixed into the mulched elephant grass at a 5% concentration during the silage process. The ingredients of the added concentrate were processed in an animal feed factory and mixed in a vertical mixer. The silage was mixed with the concentrate at the time of feeding the animals. The oil was mixed with the stored bran, and later it was fed to the animals according to their diet.

The soybean oil used in Treatment 2 was donated to the micro-entrepreneurs producing fried potatoes; it was the same brand of soybean oil used in Treatment 1. The oil for Treatment 2 was collected after being used to fry potato chips for approximately 6 h per day for 3 days, for a total of 18 h of exposure to a gas fire.

### 2.4. Slaughter of the study animals

The animals were weighed after 16 hours of fasting to determine their final mass and subsequently stunned and slaughtered according to the norms of the Regulation of the Industrial and Sanitary Inspection of Products of Animal Origin (RIISPOA) (12). First, the animals were hung by their tendons and stunned using electronarcosis, followed by bloodletting, which occurred immediately after stunning by severing the carotid artery and jugular veins. After exsanguination, the animals were skinned and eviscerated. The carcasses were then transferred to a refrigerating chamber at 6°C, for 24 h, suspended by the gastrocnemius tendon on appropriate hooks to maintain the tarsus-metatarsal articulations at a distance of 17 cm.

### 2.5. Carcass yield

The carcasses were weighed soon after slaughter (MHC = mass of hot carcass) and after 24 h of cooling (2°C) (MCC = mass of cold carcass). Carcass yield for HCY and CCY was calculated using the percentages of the hot and cold carcass masses in relation to the final mass (FP) and the loss of mass due to cooling (LMC) by the difference between the two carcass masses (13).

### 2.6. Fat surrounding the digestive system, conformation, and finishing

The omentum (rumen, reticulum, abomasum, and omasum) and mesenteric (small and large intestine) fat surrounding the digestive system were removed and weighed, and the same procedure was repeated for the perirenal fat from the kidneys (14). After being weighed, the cold carcasses were hung by the tarsus-metatarsal articulations on hooks spaced 17 cm apart and were visually evaluated in a subjective manner with respect to conformation on a scale of 1–5, ranging from excellent to poor. Using photographic standards from the European model of classification, values from 1 to 5 were also used for the variable finishing, ranging from thin to excellent.

### 2.7. Biometric measurements

The following morphological evaluations were conducted on the cold carcass: thoracic and croup widths, the circumference of the leg and arm, external and internal lengths of the carcass, and leg and arm lengths, according to the method of Osório and Osório (13).

### 2.8. Commercial cuts and tissue composition

Necks were removed by an oblique cut between the 5th and 4th cervical vertebrae, and tails were removed by a transverse incision made at the last sacral vertebra before the first of the tail. After neck and tail removal, the carcasses were sectioned longitudinally, thus obtaining two symmetrical halves. The right half of the carcass was divided into anatomical regions known as commercial cuts: shoulder, leg, neck, rack, loin, ribs/belly, and weighed separately. The percentages of each cut in relation to the left half of the carcass were calculated according to Sobrinho (15).

Using the technique described by Osório and Osório (12), the loin and leg—cuts that best predict the total tissue content of the carcass—were used from the left half of the carcass to evaluate the tissue composition through dissection. The tissues were separated into the components of muscle, bone, and fat, and then each component was individually weighed to calculate the mass and proportion, in relation to the respective cuts, and the percentage of each tissue was also calculated (12).

### 2.9. Evaluated parameters of the *Longissimus dorsi* muscle

#### 2.9.1. Determination of temperature and pH

The pH and temperature were determined using a portable penetration potentiometer, taking three readings at a depth of 5 cm inside the *Longissimus dorsi* muscle at the level of the 13th thoracic vertebra, 45 min after stunning (initial pH) and 24 h after slaughter (final pH), removing the portion of the muscle that was in direct contact with the air to avoid any possible interference due to oxidation.

#### 2.9.2. Loin area, coverage, fat thickness, and marbling

The loin eye area (LEA) was calculated using the formula:

$$\mathrm{LEA}=\left(\left(A/2\right)\times \left(B/2\right)\right)\;\pi .$$

For the area around the LEA, the thickness of the backfat covering (TBC) was measured with a stainless steel caliper, and the marbling was subjectively evaluated using the American Meat Science Association (AMSA) photographic standards (16), attributing grades from 1 to 6 (1 = traces of marbling; and 6 = abundant marbling).

#### 2.9.3. Water retention

The capacity to retain water (CRW) test was performed 48 h after slaughter using the pressure method. Samples cut into 5 g pieces were placed on filter paper between two acrylic plates, and a 10 kg mass was placed on the top plate for 5 minutes and then weighed. The quantity of water lost was calculated as the difference between the initial and final masses and then expressed as a percentage (17).

#### 2.9.4. Weight loss by cooking

In order to determine the loss of mass by cooking (LMC), the samples were cut, weighed, placed in baking pans, and heated in a preheated oven at 170°C until the internal temperature at the geometric center of the sample reached 70°C, measured by a thermocouple as described by Muller (18). The loss of mass due to cooking was calculated as the difference between the masses before and after the sample reached ambient temperature.

#### 2.9.5. Determination of objective color

The objective color was established in the L* (luminosity), a* (red/green intensity), and b* (yellow/blue intensity) spaces of the CIELAB system using a D65 illumination source, an 8° viewing angle, and a 10° observer pattern, as specified in CIE (19), using a portable colorimeter equipped with a humidity protection device.

### 2.10. Sensory analysis

Sensory analysis was conducted using 55 untrained and randomly selected beef consumer panelists. The semimembranosus muscle (without subcutaneous fat) was used, obtained from the commercial leg cut of the lambs. The samples were thawed for 24 h in a conventional refrigerator, separated according to treatments, and left at room temperature for 45 min in a 10% brine solution using a 1:1 ratio for mass. The samples were then baked in an oven to 85°C, cut into 2–3 cm strips, and served separately to the panel members at a temperature of 45–50°C on labeled disposable plates. After tasting each sample, panel members were asked to ingest water and salted crackers to remove any residual flavor from the previous sample and provide a clean palette for the next one.

For the descriptive test, a 9-point hedonic scale was used, taking into consideration the following attributes: taste (flavor sensation during chewing), texture (tenderness, juiciness, and resistance during chewing), color (visual evaluation of the sample), and aroma (odor released by the sample). The nine points of the scale consisted of: 1—extremely dislike, 2—very much dislike, 3—moderately dislike, 4—slightly dislike, 5—neither dislike nor like, 6—slightly like, 7—moderately like, 8—very much like, 9—extremely like.

### 2.11. Statistical analysis

The assumptions of normality of errors and homogeneity of variances were checked using the Cramér–von Mises and Brown-Forsythe tests, respectively, which showed no violation of these assumptions. The data were subjected to analysis of variance (ANOVA) at the 5% level of probability, and significant differences between averages were compared using Tukey’s test at the 5% level of probability. Non-parametric data were analyzed using the Kruskal-Wallis test for independent samples, and SAS software (20) was used for all statistical analyses.

## 3. Results

During the dietary supplementation phase, the animals had a similar average daily weight gain of 0.242 kg/day. There were no observed statistical differences between treatments (Table 4) for HCY, CCY, and PR. Perirenal, omental, and mesenteric fat contents were not influenced by the type of oil used in the animal feed (*p* > 0.05).

**Table 4:** Averages, standard deviation, and coefficient of variation (CV) of carcass characteristics of sheep fed with three different types of oil, Eastern Amazon.

Variable	Treatment—types of oil				*p*-value
	Soybean oil in natura	Soybean oil after frying	Palm oil	CV (%)
Mass at slaughter (kg)	41.69 ± 2.64	42.10 ± 2.52	42.93 ± 2.24	5.76	0.5615
Mass of hot carcass (kg)	20.37 ± 1.70	20.51 ± 1.67	20.63 ± 1.43	7.51	0.9406
Mass of cold carcass (kg)	19.67 ± 1.62	19.84 ± 1.60	19.82 ± 1.37	7.44	0.9658
Mass loss due to cooling (%)	3.42 ± 0.70	3.29 ± 0.42	4.22 ± 1.26	4.72	0.0589
Hot carcass yield (%)	48.81 ± 1.57	48.73 ± 2.43	48.09 ± 2.99	4.79	0.7833
Cold carcass yield (%)	47.14 ± 1.37	47.12 ± 2.26	46.21 ± 2.91	4.74	0.6210
Perirenal fat (kg)	0.353 ± 0.08a	0.437 ± 0.05b	0.516 ± 0.18b	30.77	0.0262
Omental fat (kg)	1.00 ± 0.18	1.048 ± 0.14	1.254 ± 0.40	24.99	0.1270
Mesenteric fat (kg)	0.551 ± 0.08	0.584 ± 0.15	0.683 ± 0.12	21.01	0.1905

The biometric averages of the carcasses were not influenced (*p* > 0.05) by the experimental diets (Table 5), and the same was true for the yields of the commercial cuts (shoulder, leg, neck, rack, loin, ribs/belly). Different letters in the line indicate statistical difference (*p*<0.05).

Finishing and conformation data were subjected to the non-parametric Kruskal-Wallis test. Finishing expresses the distribution and quantity of backfat on the carcass and has an important role in the protection of the carcass during storage, which serves to reduce losses due to chilling and avoid the shortening of muscle fibers due to the intense cold. In the present research, the animals from all the treatments were classified as being in Category 2 (good) for the conformation evaluation and between 2 and 3 (thin and median level of fat, respectively) with no statistical differences (*p* > 0.05), and excess perirenal fat (kg).

## 4. Discussion

The degree of unsaturation of fatty acids can have several effects on rumen metabolism. Some of the possible effects include changes in rumen fermentation, nutrient digestibility, volatile fat production, and microbial protein synthesis (21).

Thus, progress in the study of lipids in ruminant diets is a result of the concept that dietary manipulation by supplementation with lipids is a way to influence physical processes or change the fat profile of its derivatives (22). Another reason for the realization of fat supplementation in ruminant diets is the increase in its energy concentration. In addition to what was previously described, the supply of oils can influence the sensory characteristics of the product animal, as the content and composition of milk fat interfere with the flavor and texture of dairy derivatives (23) and meat, a subject that has not been widely studied.

It is important to mention that, according to the first article of Regulation No. 8, of March 25, 2004, of the Ministry of Agriculture, Livestock, and Supply (MAPA), the production, distribution, and use of products intended for feeding ruminants that contain proteins of animal origin are prohibited. This includes poultry litter, residues from pig farming, and any product containing proteins of animal origin (24). Therefore, the only frying residues that can be utilized in the feeding of ruminants are the oils derived from frying products of exclusively vegetable origin, which were used in this research.

Residual frying oil, which is subjected to high temperatures, can undergo lipid presentation, generating several complex chemical reactions that can cause nutritional damage due to the partial ingestion of essential unsaturated fats such as linoleic and linolenic acids and other lipids such as vitamin A, carotenoids, and tocopherols. The production of peroxides can also irritate the intestinal mucosa, leading to diarrhea and reduced nutrient absorption (25). Oxidized products also significantly alter their organoleptic properties, which can reduce food consumption by animals (26).

During the frying process, compounds with carcinogenic properties, such as polyaromatic hydrocarbons, may still be formed, and further studies on the frequent or continuous use of residual frying oil are needed. However, its use in the feeding of finishing ruminants, and therefore for a limited period of time, has demonstrated an effect similar to that of the use of soybean oil in natura on animal consumption, digestibility, and performance (Oliveira et al., citing authors who have worked with the oil).

The results for PCQ, PCF, and HCY, in addition to CCY and cooling losses (CL), may have occurred due to the similarity of the sheep masses at slaughter, considering that these variables are highly related when carcass yield is not affected (27). It is important to highlight the similarities in slaughter mass and the identical pre-slaughter management for all three treatments. According to Restle et al. (28), carcass yield is influenced by, among other variables, the number of hours of fasting to which the animals are subjected, the type of diet, and the animals’ genetic makeup. The CL data in the current work are similar to those of Sobrinho (15), who analyzed slaughterhouse data and observed that the average mass loss due to cooling in sheep was 4%.

The inclusion of fat in the diet, through the three types of oil used in the current study, could have accentuated the deposition of fat in the carcasses, thus providing an adequate quantity of backfat and reducing the losses due to cooling. In all treatments, the HCY and CCY values are in agreement with those normally found in the literature for this species of sheep (above 40%), similar to those observed by Cunha et al. (29), who found 47.6% for HCY and 46.6% for CCY in Santa Inês lambs, and by Garcia et al. (30), who found 47.2% for HCY and 45.4% for CCY em sheep ½ Santa Inês x ½ Dorper.

Regarding the parameter “fats,” the data of the current study differ from those observed by Alves et al. (31), where the fat content from Santa Inês lambs that were fed diets that had different energy levels showed a large variation as a function of the nutritional level. The reduction in internal fat should be seen as a positive result because, according to Ferreira et al. (32), it is not used for human consumption and is considered a waste of food energy, being, therefore, undesirable due to the lower metabolic rate of the adipose tissue, which, therefore, increases the energy required for maintenance.

Carcass biometric measurements were not influenced by the treatments, and this is reflected in the similar growth of the animals and the absence of differences in the total mass gain, which resulted in carcasses with similar size measurements between treatments.

The absence of an effect of diets on the yield of commercial cuts has been reported in the literature (33). On the other hand, Cartaxo et al. (34) observed that a diet with a higher concentration of energy, promoted by the inclusion of 2% soybean oil, favored a larger yield for the leg cut, the portion that has a greater deposit of muscle mass.

As for the composition of the tissue, there was no influence on these variables, and this is probably due to the fact that all the diets had high energy levels and the animals were all of the same age. Fushuro-Garcia et al. (35) reported that the shoulder and the leg represented more than 50% of the carcass and that these cuts were the most predictive of the total tissue content of a carcass.

Cartaxo et al. (34) showed that the inclusion of 2% soybean oil in the diet promoted a larger LEA in the sheep carcass, with a value of 12.42 cm^2^ for the LEA, inferior to those obtained in the current study, where the inclusion of 4% of three different oils was used. For sheep carcasses, there is still no standard value for the minimum thickness of the backfat that determines an ideal finishing condition; however, Sobrinho (15) affirms that the average thickness of the backfat varies between 2 and 5 mm. In the current study, the values found for the thickness of the backfat can therefore be considered adequate, with values ranging from 1.83 to 2.33 mm.

The marbling of the meat showed no differences between treatments, but it is important to highlight that the development of fat interspersed in the meat occurs when the animal is gaining mass at an elevated rate, or when it is of advanced age since the last fat that is deposited is the first to be used in metabolic activities when the animal is under alimentary restriction (36).

The average final pH found in the current work was 5.74, a value considered adequate (37, 38). It is important to emphasize that lambs stressed prior to slaughter present a lower reserve of muscle glycogen and final pH values above 5.8 (39).

The loss of mass due to cooking and the water retention capacity did not present results similar to those of Leão (37). The CRW had higher values, but that was within the accepted limits, which indicates that the meat did not present abnormalities, most likely due to the pH values being within normal limits.

Even without differences (*p* > 0.05) between the experimental diets for the variable color, where L*, a*, and b* (Table 5), the meat had a clearer appearance (53.64), lighter (53.64), less red (30.32) and paler appearance (17.47), when compared to that of lambs fed with different levels of incorporation of residual cooking oil from frying, as evaluated in the work of Pinheiro (38). In the present study, the intense red color may have been influenced by the pH as a function of the energy level of the diet. According to Missio et al. (39), the level of concentrate feed in the diet influences the color of the meat and shows a linear increase, meaning that increasing the content of concentrate feed improves the color of the meat.

**Table 5:** Averages, standard deviation, and coefficient of variation (CV) of biometric measurements and of mass of commercial cuts of carcasses of sheep fed with three different types of oil, Eastern Amazon.

Variable	Treatment—types of oil				
	Soybean oil in natura	Soybean oil after frying	Palm oil	CV (%)	*p*-value
Thoracic width (cm)	23.05 ± 0.77	23.39 ± 0.48	23.44 ± 1.36	3.96	0.2440
Croup width (cm)	21.04 ± 1.32	21.03 ± 1.20	21.30 ± 0.75	5.11	0.8498
Thoracic depth (cm)	36.72 ± 1.50	36.17 ± 1.25	36.85 ± 1.32	3.87	0.5324
Croup perimeter (cm)	55.25 ± 2.45	53.17 ± 2.65	52.72 ± 1.93	4.72	0.0719
Leg perimeter (cm)	17.79 ± 1.60	17.51 ± 0.93	17.83 ± 1.15	6.87	0.8418
Arm circumference (cm)	17.68 ± 0.56	18.24 ± 0.93	18.04 ± 0.56	3.99	0.2440
Outer length of the carcass (cm)	75.97 ± 2.91	76.54 ± 1.59	76.11 ± 2.71	3.13	0.8763
Inner length of the carcass (cm)	71.63 ± 3.25	72.15 ± 2.39	70.52 ± 2.31	3.73	0.4314
Leg length (cm)	14.90 ± 1.89	15.11 ± 0.86	14.90 ± 2.10	10.97	0.9552
Arm length (cm)	12.60 ± 1.65	12.33 ± 1.27	12.00 ± 1.00	10.61	0.6388
Shoulder (kg)	1.665 ± 0.15	1.734 ± 0.29	1.830 ± 0.18	13.13	0.3194
Leg (kg)	2.884 ± 0.24	3.070 ± 0.20	3.054 ± 0.15	8.14	0.2093
Neck (kg)	0.596 ± 0.12	0.640 ± 0.11	0.647 ± 0.10	17.54	0.4592
Rack (kg)	1.835 ± 0.28	1.766 ± 0.24	1.774 ± 0.26	14.00	0.8277
Loin (kg)	0.755 ± 0.16	0.751 ± 0.11	0.778 ± 0.15	18.00	0.9099
Rib/belly (kg)	1.867 ± 0.18	1.853 ± 0.16	1.970 ± 0.12	8.26	0.2265

The leg was the cut with the highest yield, representing about 30% of the left half of the carcass, and also had the largest percentage of meat taken from the carcass. This cut is also considered the most valuable part of the sheep. There was no effect of oil type on tissue composition (*p* > 0.05) (Table 6).

**Table 6:** Averages, standard deviation, and coefficient of variation (CV) of tissue composition muscle, bone, and fat of the leg and loin cuts of sheep fed with three different types of oil, Eastern Amazon.

Variable		Treatment—types of oil				
		Soybean oil in natura	Soybean oil after frying	Palm oil	CV (%)	*p*-value
Muscle						
Loin	Kg	0.436 ± 0.11	0.435 ± 0.06	0.429 ± 0.05	17.65	0.9788
	%	60.05	57.77	58.85		
Leg	Kg	1.742 ± 0.17	1.788 ± 0.33	1.829 ± 0.28	14.88	0.7976
	%	63.05	63.45	62.87		
Bone						
Loin	Kg	0.163 ± 0.58	0.186 ± 0.04	0.167 ± 0.04	27.00	04783
	%	22.45	24.70	22.91		
Leg	Kg	0.584 ± 0.12	0.590 ± 0.13	0.650 ± 0.09	19.31	0.4334
	%	21.14	20.94	22.34		
Fat						
Loin	Kg	0.127 ± 0.05	0.132 ± 0.04	0.133 ± 0.05	33.70	0.9468
	%	17.49	17.53	18.24		
Leg	Kg	0.437 ± 0.06	0.440 ± 0.10	0.430 ± 0.10	20.38	0.9556
	%	15.82	15.61	14.78		

The use of different types of oils in the diets did not influence (*p* > 0.05) the area of the rib eye or the thickness of the backfat (Table 7). The marbling of the meat represented the quantity of intramuscular fat, there was no difference between treatments (*p* > 0.05) for this variable, and all animals in all treatments were classified as being in Category 2 (light marbling). Furthermore, there was no treatment effect observed for the pH or initial and final carcass temperatures.

**Table 7:** Averages, standard deviation, and coefficient of variation (CV) of parameters of the *Longissimus dorsi* muscle of sheep fed with three different types of oil, Eastern Amazon.

Variable	Treatment—types of oil				
	Soybean oil in natura	Soybean oil after frying	Palm oil	CV (%)	*p*-value
Loin eye area (cm^2^)	15.60 ± 1.32	16.33 ± 2.71	16.12 ± 1.99	12.64	0.7470
Backfat thickness (mm)	1.83 ± 0.35	2.33 ± 0.89	2.33 ± 0.97	35.63	0.2948
Initial pH	6.08 ± 0.26	5.93 ± 0.38	5.90 ± 0.38	5.68	0.4847
Final pH	5.77 ± 0.17	5.69 ± 0.11	5.76 ± 0.16	2.60	0.5353
Initial temperature	31.10 ± 1.62	30.25 ± 1.60	30.60 ± 1.98	5.61	0.5843
Final temperature	11.73 ± 1.73	11.46 ± 2.00	10.75 ± 1.73	15.97	0.4726
Mass loss due to cooking (%)	7.26 ± 1.01	6.57 ± 1.35	6.42 ± 0.71	16.04	0.2197
Water retention capacity (%)	0.49 ± 0.16	0.47 ± 0.12	0.52 ± 0.10	25.98	0.7294
L^*^ (luminosity)	55.02 ± 2.05	52.97 ± 2.86	52.94 ± 5.28	6.81	0.3992
a^*^ (red/green intensity)	30.79 ± 3.08	31.48 ± 3.01	28.69 ± 4,04	11.52	0.2155
b^*^ (yellow/blue intensity)	17.57 ± 1.19	18.09 ± 1.57	16.77 ± 2.09	9.66	0.2559

CV, coefficient of variation.

Associated with the yield and important in the preparation for consumption is the juiciness, and in this study there were no statistical differences found for this variable between treatments. In the evaluation of sensory attributes, none of the studied characteristics showed a treatment effect (*p* > 0.05) (Table 8). The ratings attributed to the taste of the meat were not influenced by the different types of oil used in the diets. However, the scores given by the panelists for these attributes indicate that the meat from these animals was highly palatable.

**Table 8:** Average values attributed by panelists to meat samples from sheep supplemented with three different types of oil.

Variable	Treatment—types of oil				*p*-value
	Soybean oil in natura	Soybean oil after frying	Palm oil	CV (%)
Color	6.27	6.96	6.85	2.22	0.109
Taste	7.09	7.00	7.18	3.06	0.531
Texture	6.13	7.01	7.10	4.12	0.692
Aroma	5.61	6.89	6.76	2.24	0.724

With respect to the evaluation of the sensory attributes of the meat, none of the variables studied was influenced by the treatments, and this result favors the use of any of the three oils in the feed of sheep because factors such as appearance (color), taste, tenderness, and aroma (smell) of the meat positively influence the reaction of the consumer. However, these attributes can vary based on age, sex, breed, and feed type. The aroma (smell) of the meat did not differ (*p* > 0.05) between treatments with different types of oil incorporated into the diet. In this study, the content of fat in the muscle did not vary between the diets (4%), which could explain the similarity between the ratings obtained in the sensory qualitative analysis, which varied between 5.61 and 6.89. According to Madruga et al. (40), the aroma of the meat is directly related to the fat content present in the muscle.

## 5. Conclusion

After assessing the influence of the diet of lambs fed with different types of oils on the quantitative and qualitative characteristics of the carcass and meat, it was concluded that the use of soybean oil in natura, soybean oil residues after use in frying, and palm oil, added to the sheep diet did not alter any of the evaluated carcass and meat characteristics. This result favors the use of any of these three oils as a supplement to the sheep diet since the three oils provide good carcass and meat characteristics. In this context, the use of residual soybean oil from frying, or palm oil, represents interesting alternatives in the face of environmental, social, and economic issues.

## Data availability statement

The raw data supporting the conclusions of this article will be made available by the authors, without undue reservation.

## Ethics statement

The experiment was conducted in compliance with the ethical norms established by the Committee for the Ethical Treatment of Animals in Experimentation of the Federal Rural University of the Amazon (UFRA), and was approved by the same committee under protocol number 005/2013.

## Author contributions

TC, AR and JL-J: experiment design. TC, JS, CF, AS, AR, EM, and JL-J: experiment execution TC, JS, CF, AS, AR, EM, JB, VC, AB, WS, AS, and JL-J: data curation. WS and JL-J: formal analysis and original writing. All authors edited and approved the final manuscript.

## Funding

This study was partially funded by the Federal University of Pará and the Coordenação de Aperfeiçoamento de Pessoal de Nível Superior (CAPES) Brasil. This study also received financial support for the publication fee from the Pró-Reitoria de Pes-quisa e Pós-Graduação (PROPESP/UFPA—Announcement—02/2023).

## Conflict of interest

The authors declare that the research was conducted in the absence of any commercial or financial relationships that could be construed as a potential conflict of interest.

## Publisher’s note

All claims expressed in this article are solely those of the authors and do not necessarily represent those of their affiliated organizations, or those of the publisher, the editors and the reviewers. Any product that may be evaluated in this article, or claim that may be made by its manufacturer, is not guaranteed or endorsed by the publisher.
